# Thermal and Dielectric Properties of Cyanate Ester Cured Main Chain Rigid-Rod Epoxy Resin

**DOI:** 10.3390/polym13172917

**Published:** 2021-08-30

**Authors:** Chi-Ping Li, Chih-Min Chuang

**Affiliations:** 1 Department of Chemical Engineering, National United University, Miaoli 36063, Taiwan; 2 Institute of Nuclear Energy Research, Longtan, Taoyaun 32546, Taiwan; cmchuang@iner.gov.tw

**Keywords:** epoxy resin, liquid crystal, cyanate esters, thermal properties, dielectric properties

## Abstract

Thermal and dielectric properties of rigid-rod bifunctional epoxy resin 4,4-bis(2,3-epoxypropoxy) biphenyl epoxy (BP) and commercial epoxy resin diglycidyl ether of bisphenol A (DGEBA) were studied using differential scanning calorimeter (DSC), thermogravimetric analyzer (TGA), dynamic mechanical analyzer (DMA), thermal mechanical analyzer (TMA) and dielectric analyzer (DEA). These two epoxies were cured with cyanate ester hardener 2,2’-bis(4-cyanatophenyl) propane (AroCy B10). The BP/B10 system consisting of a rigid-rod structure exhibited better thermal properties than the DGEBA/B10 system with a flexible structure. Anisotropic BP/B10 (2:1) had the highest 5% weight loss temperature, the highest amount of residue and a smaller thermal expansion coefficient than the commercial DGEBA/B10 system. The BP/B10 system, which cured at the LC phase temperature, had higher Tg than the commercial DGEBA/B10 system, as found from dynamic mechanical analysis. The BP/B10 system also demonstrated better dielectric properties than the commercial DGEBA/B10 system when enough curing agent was provided.

## 1. Introduction

Epoxies (EP) have been widely used in industrial applications, such as for coatings, adhesives, composite materials, electronic components, etc., due to their excellent characteristics such as easy processing, high mechanical strength, low shrinkage, resistance to chemical solvents, heat resistance, adhesion and good electrical insulation [1,2,3,4,5,6,7,8,9,10]. DGEBA is the most studied epoxy resin that has been widely used in various industrial applications, such as paints, coatings and adhesive applications, due to its good mechanical and thermal properties, low curing shrinkage and good chemical resistance. Another reason for the popularity of DGEBA is its low cost. However, there are some serious drawbacks, such as brittleness or low fracture energy, significantly restricting its extensive utilization [11,12]. Other epoxies, consisting of an aromatic rigid-rod backbone structure, are well-known to exhibit outstanding thermal and mechanical properties. A great deal of these types of epoxies also have liquid crystal (LC) behavior [13,14]. Rigid-rod thermoset epoxies have been synthesized for use as encapsulation materials because of their good performance. Some of them retain the LC behavior after being cured with the curing agent [15,16,17]. Su et al. synthesized an epoxy (BP) using biphenol mesogens and epichlorohydrin that displayed high glass transition temperature, low thermal expansion coefficients, high dielectric strengths and low dielectric loss after curing [13,18,19]. Su et al. showed that BP/sulfanilamide (SAA) displayed LC phase after curing, which resulted in high glass transition temperature and a low thermal expansion coefficient [20].

Cyanate ester (CE) resins possess excellent dielectric properties, thermal resistance and good flame-retardancy [21,22,23,24,25,26]. CE belong to a family of monomers with cyanate end groups (-O-C≡N-) which provide good thermal degradation temperature due to forming an aryl cyanurate network through a trimerization reaction. The mechanism of the cyanate ester reacting with epoxy first produces the alkyl cyanurate, and alkyl isocyanurate is formed after rearrangement, then cycle oxalolidinone is formed after reacting with epoxy [27,28,29]. Those high-performance EP/CE resins can be used in electronic packaging encapsulants and printed circuit boards due to their great thermal and dielectric properties [30,31,32,33,34,35]. Krishnadevi and Selvaraj used DGEBA/B10 combined with flame-retardant and functionalized rice husk ash, which showed good dielectric properties and thermal stability [36]. Wu et al. integrated tetra methyl biphenyl epoxy (TMBP) with B10, which also exhibited great dielectric properties and thermal stability [37]. Ariraman et al. presented a combination of DGEBA/CE and terminated polydimethylsiloxane to obtain a thermally stable and low dielectric constant material [38]. Yu et al. added liquid crystalline epoxy resin into DGEBA/B10, which demonstrated an improvement of glass transition temperature, thermal expansion coefficient and mechanical properties [39]. Liang et al. demonstrated that lower molecular weight EP in the DGEBA/CE system had better thermal, moisture absorption and mechanical properties [40]. Wu et al. reported that DGEBA/B10 mixed with glutaric anhydride (GA) and zinc (II) acetylacetonate (ZAA) exhibited high initial thermal decomposition temperatures of 337–353 °C. The EP/CE/GA/ZAA system had thermal self-healing behavior resulting from the transesterification and possible reactions between the residual cyanate ester and epoxy groups, and a 78–83% fracture toughness recovery of the hybrid can be achieved after the first healing rate of 200 °C/2 h [41]. Wang et al. declared that diglycidyl ether of bisphenol F (DGEBF)/B10 mixed with phenolphthalein poly(ether sulfone) displayed higher fracture toughness and impact strength [42]. Wang et al. highlighted that the glass transition temperature, thermal stability and moisture absorption were found to increase with the epoxy functionality when naphthalene-containing epoxy resins were cured with B10 [43]. Ho et al. pronounced that DGEBA and 2-(6-oxido-6H-dibenz(c,e)(1,2)-oxaphosphorin-6-yl)-1,4-benzenediol (DOPOBQ) cured with various CE exhibited higher glass transition temperatures, better dimensional stability and better thermal stability [44].

Our previous work [45] studied the BP/B10 system but did not look at the details of the curing process and try to keep the LC (anisotropic) phase after curing. In this research, liquid crystalline EP (BP) was cured using CE (B10) as a curing agent and formed anisotropic phase in the materials. The thermal and dielectric properties of the BP/B10 and DGEBA/B10 systems were investigated in this research. The novelty and significance of this work is to study how to maintain the LC phase in the cured epoxy resin using CE as a curing agent and to study the improvement of the thermal and dielectric properties. Few studies keep LC phase in epoxy through applying CE. The methodology of this research uses DSC, TGA, DMA and TMA to perform the thermal analysis. The DEA was used to carry out the dielectric analysis.

## 2. Materials and Methods

### 2.1. Materials

**Figure 1:**
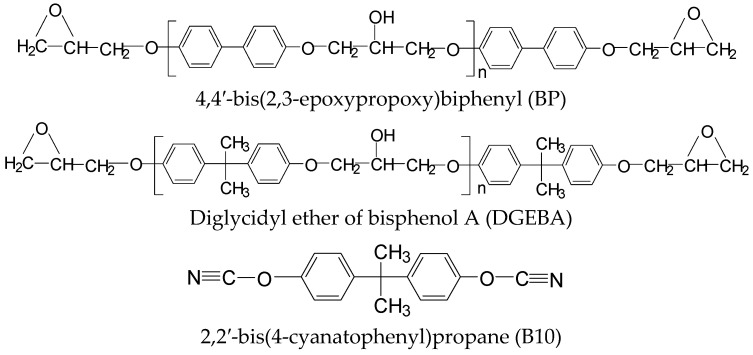
The chemical structures of epoxy resins (BP and DGEBA) and the curing agent (B10).

The BP with an epoxy equivalent weight of 180 was synthesized according to previous literature [18]. The DGEBA was obtained from Dow Chemical Company (Midland, MI, USA) with an epoxy equivalent weight of 185.3. The curing agent B10 was purchased from Ciba-Geigy (Tokyo, Japan). The structures of the epoxy resin and curing agent are shown in Figure 1. The catalyst copper(II) acetylacetonate was purchased from Aldrich (St. Louis, MO, USA).

### 2.2. Preparation of Cured Epoxy

One TA instrument, DSC-10, was used to determine the curing conditions of the EP/CE system. This experiment uses different epoxy resins (BP and DGEBA) and a curing agent (B10) in different ratios (2:1 and 1:1), and 0.1% of the total weight of the catalyst, copper(II) acetylacetonate, which was grounded before adding to the epoxy resin. The mixture was heated to 130 °C to melt and then stirred. After the mixture was mixed well, it was poured into a preheated mold for the curing reaction.

#### 2.2.1. DGEBA/B10 System

The required equivalents of the epoxy resin (DGEBA) and the cyanate ester curing agent (B10) were precisely weighed. Then, 0.1% of the total weight of the catalyst was mixed with B10, ground with a grindstone, then the epoxy resin was added and heated up to 130 °C. After the sample was completely melted, the mixture was stirred thoroughly and poured into the preheated mold. The curing condition was as follows: curing at 130 °C for 6 h, at 175 °C for 6 h and then at 200 °C for 6 h, all at a heating rate of 10 °C per minute when raising the temperature.

#### 2.2.2. BP/B10 System

The mixing method was the same as above. The curing condition of normal BP/B10 (2:1) was: curing at 130 °C for 6 h, at 150 °C for 6 h, at 175 °C for 6 h and at 200 °C for 6 h, all at a heating rate of 10 °C per minute when raising the temperature. In order to preserve the anisotropic properties in the BP/B10 (2:1) system after curing, the curing condition occurred under vacuum and curing at a temperature (150 °C) where BP is at a LC phase for 16 h. Then, the temperature was increased to 220 °C at a heating rate of 1 °C per minute to cure for 6 h. This BP/B10 (2:1) is addressed as anisotropic BP/B10 (2:1). The BP/B10 (1:1) had the same curing condition.

### 2.3. Morphological Observation of the Epoxy Resin

The BP/B10 mixture was placed on a hot plate. A polarized optical microscope (POM, NIKON, ECLIPSE ME600, Tokyo, Japan) and a V8 camera were used to observe and record the morphology changes of the sample during different curing processes.

### 2.4. XRD Measurement

The X-ray diffraction measurement was obtained from a Rigaku D/Max-VIII instrument at 2°/min, 3 to 30°, at 40 KV, 30 mA.

### 2.5. Thermal Properties

The TA instrument DSC-10 (5 °C/min in 50 mL/min N_2_) was used to determine the Tg of the cured resins. The Du Pont TGA 951 thermal gravimetric analyzer (TGA) (10 °C/min in 50 mL/min N_2_) was used to study the weight loss with increasing temperature. The Du Pont 9900 983 DMA (5 °C/min in air, 1 Hz, 20 μm amplitude) was used to identify the change in modulus and damping properties. The TA instrument TMA 2940 (10 °C/min in air) was used to measure the coefficient of thermal expansion. 

### 2.6. Dielectric Properties

The TA instrument DEA 2970 (10, 100, 1000 and 10,000 Hz, in 50 mL/min N_2_) was used to study the dielectric properties of cured epoxy resins.

## 3. Results and Discussion

### 3.1. Morphological Observation

**Figure 2:**
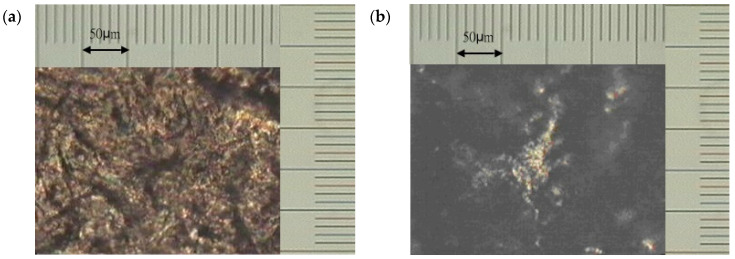

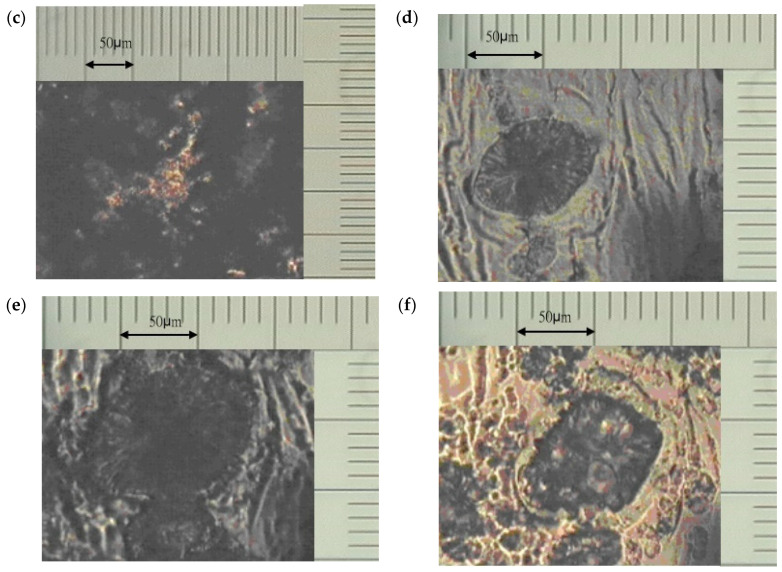
Polarized optical microscope (POM) image of (**a**) BP/B10 (2:1) 150 °C/1 h, 200×. (**b**) BP/B10 (2:1) 150 °C/5 h, 200×. (**c**) BP/B10 (2:1) 150 °C/5 h, ramp 10 °C/min to 210 °C, 200×. (**d**) BP/B10 (2:1) 160 °C/1 h, 500×. (**e**) BP/B10 (2:1) 160 °C/5 h, 500×. (**f**) BP/B10 (2:1) 160 °C/5 h, ramp 10 °C/min to 210 °C, 500×.

Figure 2a shows that the BP/B10 (2:1) mixture was cured on a hot plate at a constant temperature of 150 °C for 1 h, and the resulting anisotropic morphology was observed with a polarizing microscope (POM). Figure 2b shows that the mixture was cured at a constant temperature of 150 °C for 5 h and anisotropic textures were formed. Figure 2c shows the configuration after being cured at 150 °C for 5 h and then heated to 210 °C at a rate of 10 °C/min. In the figure, each grid of the scale is 10 μm.

Figure 2d shows that the BP/B10 (2:1) mixture was cured on a hot plate at 160 °C for 1 h, and the resulting morphology was observed with a POM. Figure 2e shows that the mixture was cured at 160 °C for 5 h at a constant temperature, and its anisotropic texture is also shown here. Figure 2f shows the texture after being cured at 160 °C for 5 h and then heated to 210 °C at a rate of 10 °C/min. The LC phase grew in certain directions and evolved an anisotropic crystallization. The formation of micro-sized LC phase with fibrous anisotropic texture showing crystalline morphology of BP cured with a curing agent can also be seen in our previous work [46].

The observation results showed that a constant temperature of 150 °C produces optical anisotropic texture within a few minutes, while a constant temperature of 160 °C produces optical anisotropic texture in about an hour. The textures of the two were different. At a constant temperature of 160 °C, it was spherical and had a filamentous structure. The domain generated at a constant temperature of 150 °C was much denser than that at a constant temperature of 160 °C. Combining this result and the DSC data [18] that BP exhibits LC phase at 150 °C, the curing condition at the first stage which can maintain LC phase was 150 °C.

### 3.2. XRD Analysis

**Figure 3:**
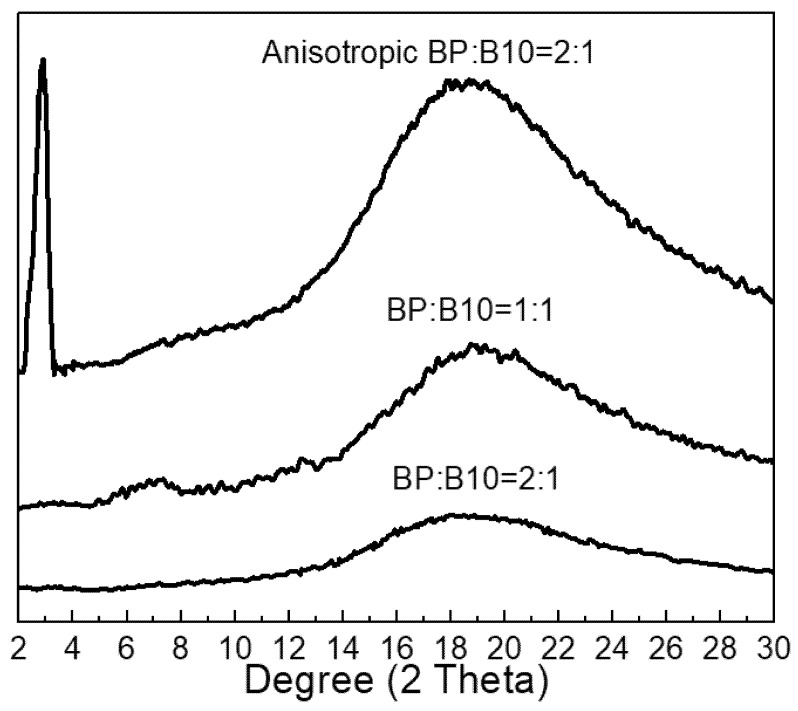
X-ray diffraction patterns of cured rigid-rod epoxy resins.

By using DSC and polarized microscopy, we observed a smectic LC phase in the range of 128–153 °C for BP epoxy [20]. The anisotropic BP/B10 (2:1) sample exhibited partially opaque features, and BP/B10 (1:1) showed much less opaque but not clear features. The BP/B10 (2:1) showed less clear features. The opaque features indicated that there was a crystalline structure in the anisotropic BP/B10 (2:1). The X-ray analysis was used to examine the crystalline phase of the BP/B10 system. Anisotropic BP/B10 (2:1) had a crystalline structure, as shown in Figure 3. The sharp diffraction peak at 2θ = 3°, and d spacing is equal to 29.4 Å. Another broad diffraction peak at 2θ = 16–24°, and d spacing is equal to 4–5 Å. Our previous work [20] demonstrated that smectic structure is preserved in the BP/SAA system, with the same broad peak in the XRD, which indicated that anisotropic BP/B10 (2:1) had smectic phase. The X-ray diffraction peak indicating smectic LC phase can also be observed in the literature [47]. The highly ordered structure of the anisotropic BP/B10 (2:1) system may be responsible for the good thermal stability and lowest CTE value of all samples.

### 3.3. TGA Analysis

The TGA scanning curves were measured at a heating rate of 10 °C/min under N_2_. In the TGA plot, the beginning temperature of decomposition (the temperature at which the weight loss reaches 5%) and the char yield at 350, 400, 450 and 500 °C were obtained. The plots were used to differentiate the temperature, and thus the fastest decomposition rate and the fastest decomposition rate temperature were obtained.

**Figure 4:**
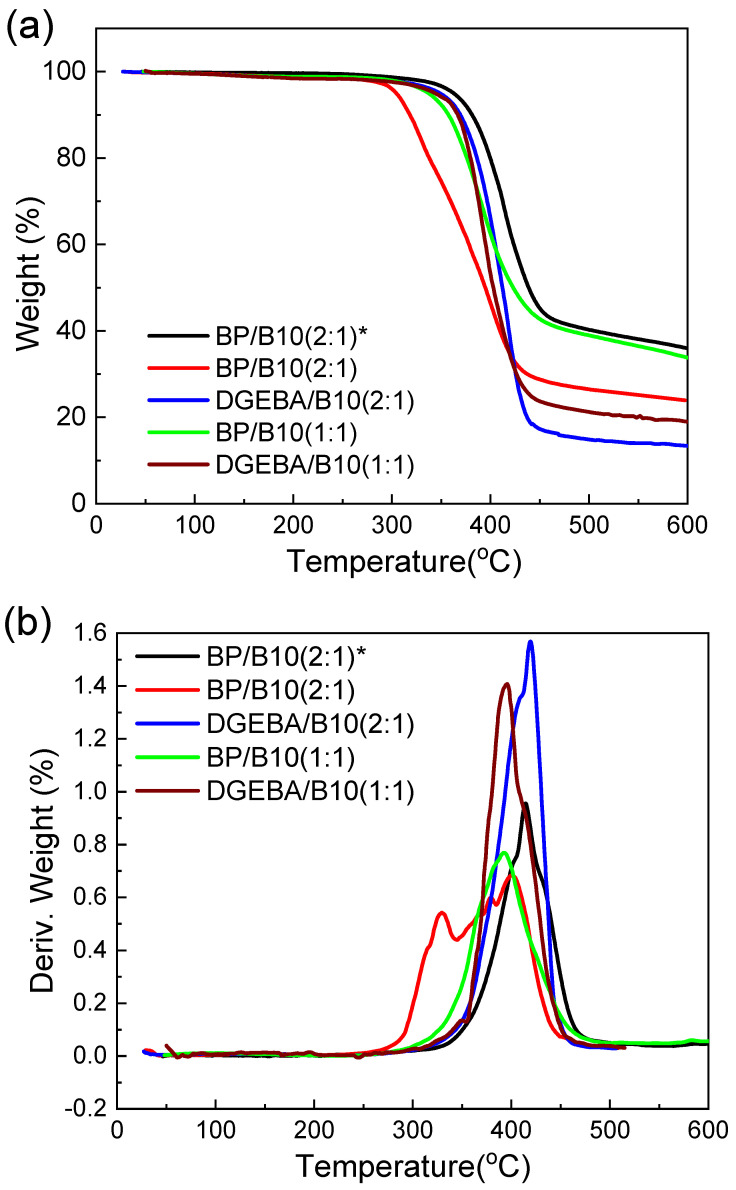
(**a**) Thermogravimetric analysis of cyanate ester (B10)-cured epoxy resin, (**b**) derivative weight versus temperatures of B10-cured epoxy resin (*: anisotropic).

As seen from Table 1 and Figure 4a, the temperature of 5% weight loss of anisotropic BP/B10 (2:1) was the highest, and the amount of decomposition residue was also the highest. This is because of the LC structure in the anisotropic sample and the enhanced thermal stability. The sample of BP/B10 (2:1) had the lowest temperature of 5% weight loss. Although the 5% weight loss temperature of DGEBA/B10 (2:1) was higher than that of BP/B10 (2:1), the amount of decomposition residue was the lowest at 450 and 500 °C. This is due to the flexible structure of DGEBA, which can easily crosslink and formed a denser network than the normal BP sample after curing, and it displayed high thermal stability at the beginning of high temperatures, but it decomposed fast and retained fewer residues after reaching a higher temperature because it does not have a rigid-rod structure. Those phenomena agree with our previous research [45]. The temperature of 5% weight loss of anisotropic BP/B10 (2:1) was 15 °C higher than the anisotropic BP/DDS (1:1) system [46], although anisotropic BP/DDS (1:1) had a higher curing temperature and formed LC phase much easier. This may be because BP/B10 (2:1) was cured at LC temperature for a long time to fix the LC phase, and was thus not easy to crack. The amount of decomposition residue in BP/B10 (1:1) was higher than that of DGEBA/B10 (1:1). This BP had more rigid-rod molecules than DGEBA. The fastest decomposition rate of DGEBA/B10 (1:1) was the largest due to a lack of rigid-rod chains.

From Table 2, anisotropic BP/B10 (2:1) had the highest and fastest decomposition rate temperature because of the existence of LC phase, and DGEBA/B10 (2:1) had the highest and fastest decomposition rate due to flexible main chains of DGEBA. Figure 4b shows that anisotropic BP/B10 (2:1) and BP/B10 (2:1) had two-stage decomposition, which may be from the decomposition of two portions in the sample, such as linear epoxy resin and cyanate ester network structure. This may be because the curing agent was not enough, and more uncured linear portions existed in BP/B10 (2:1).

From Figure 4a, it can be seen that anisotropic BP/B10 (2:1) had a higher decomposition residue than BP/B10 (1:1), which was due to the fact that there were more ordered rigid-rod structures in anisotropic BP/B10 (2:1) and it did not crack easily at high temperatures. The more LC phase in the sample, the more residue at high temperatures, as can also be seen in the literature [39]. In anisotropic BP/B10 (2:1), the fastest decomposition rate was larger, because BP/B10 (1:1) had more cyanate resin, and it formed a denser network structure (Table 2).

It is speculated that because anisotropic BP/B10 (2:1) had a longer curing time and had time to form a more stable network structure, the 5% weight loss temperature and the amount of decomposition residue are higher. The DGEBA molecular chain was relatively soft, although it was easy to move and crosslink, and the network structure was dense, but it was easier to break and crack at high temperatures, so the fastest cracking rate was higher, and the rigid-rod chain structure of the BP system was less easy to crack into small molecules.

### 3.4. DMA Analysis

DMA has been widely used to test the phenomenon of molecular motion in materials. Since thermoset epoxy resin will form a three-dimensional cross-linked network structure, molecular motion is restricted, so the degree of cross-linking will affect the polymer. Therefore, Nilsen [48] proposed the empirical equation of glass transition temperature (Tg) and crosslinking density (1/${\bar{M}}_{c}$):

(1) $$T_{g}-T_{g}^{0}≅\frac{3.9\times 10^{4}}{{\bar{M}}_{c}}$$

where, ${\bar{M}}_{c}$: the average molecular weight between the two cross-linking points, Tg^0^: glass transition temperature of un-crosslinked polymer and Tg: glass transition temperature of crosslinked polymer.

The Equation (1) can be used to illustrate the relationship between Tg and crosslinking density, that is, the larger the crosslinking density of the network structure, the higher the Tg.

**Figure 5:**
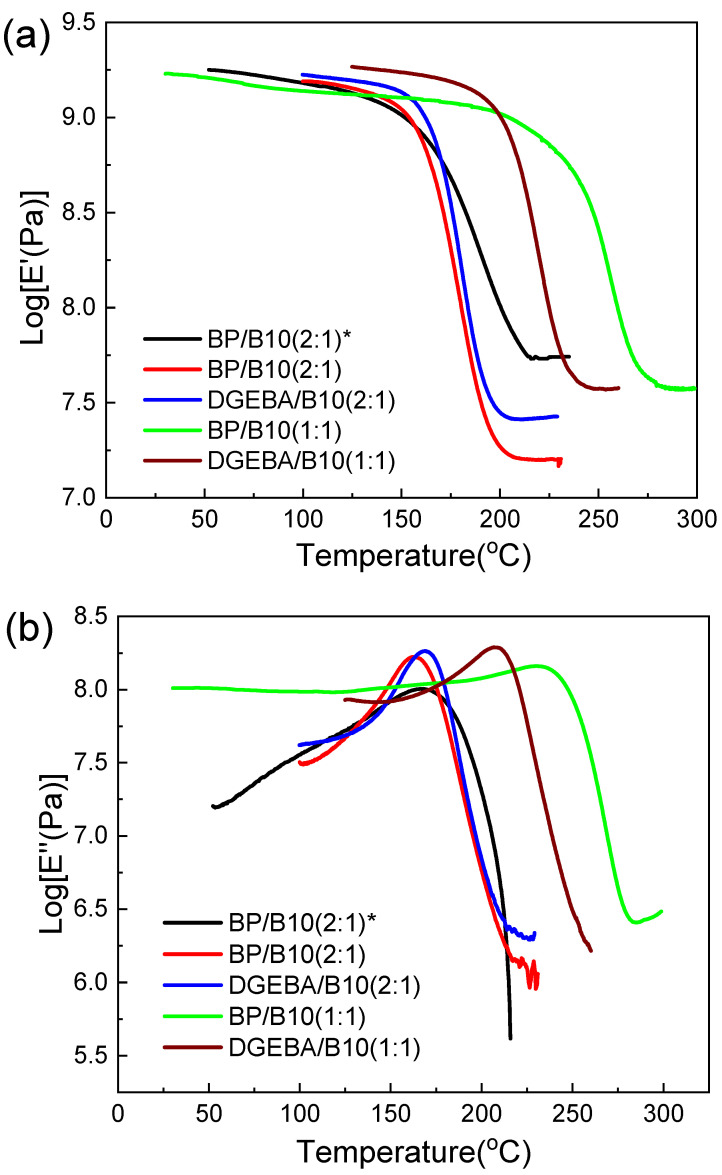

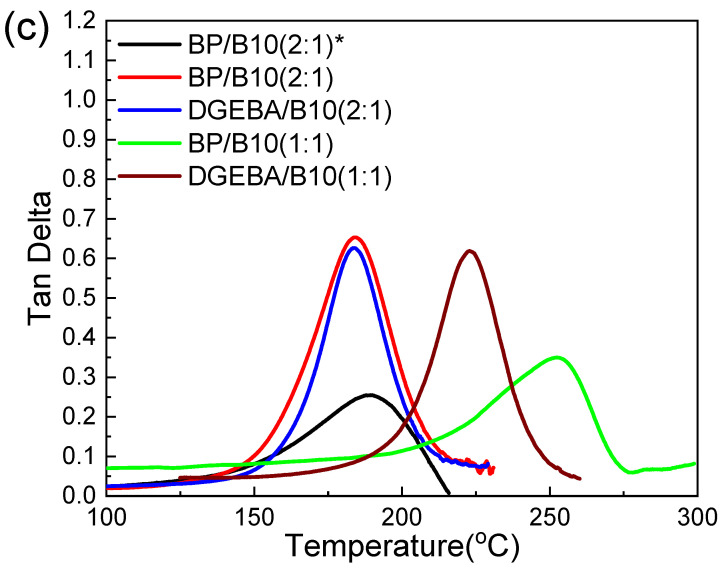
(**a**) Dynamic mechanical analysis (storage modulus), (**b**) dynamic mechanical analysis (loss modulus) and (**c**) dynamic mechanical analysis (tan delta) of cyanate ester (B10)-cured epoxy resin in epoxy:B10 = 2:1 (*: anisotropic).

It can be seen from Table 3 and Figure 5a–c, that anisotropic BP/B10 (2:1) had a higher Tg, tan δ was the lowest and the structure was the hardest. Due to its longer curing time at LC phase temperature of BP, it formed a relatively stable network structure, so higher modulus was stored at a higher temperature and Tg was higher. The results of normal BP/B10 and DGEBA/B10 systems agreed with our previous work [45]. For the same reason, the Tg of the BP/B10 (1:1) sample was also higher than that of DGEBA/B10 (1:1), and the network structure was also harder, as seen in Figure 5b. The Tg of anisotropic BP/B10 (2:1) was only 12.9 °C lower than anisotropic BP/DDS (1:1) [46], although the curing temperature of anisotropic BP/DDS (1:1) was much higher. The Tg of anisotropic BP/SAA (1:1) [20] was 29 °C higher than anisotropic BP/B10 (2:1) but 34 °C lower than BP/B10 (1:1), because more curing agent was provided in the BP/B10 system and cured at the LC temperature to improve crosslinking density and structure, thereby obtaining a better Tg.

### 3.5. TMA Analysis

For thermosetting epoxy resin processability, dimensional stability is very important. Good dimensional stability means that the product is not easily deformed and damaged by external forces. This experiment used TMA to detect the coefficient of thermal expansion (CTE) of rigid-rod epoxy resin after curing, and compared it with the commercial DGEBA.

Table 4 shows the CTE of the crosslinked structures of epoxy resin and cyanate ester after curing. They respectively represent the CTE of different crosslinked structures from 50 °C to Tg (α1) and higher than Tg, to about 50 °C above Tg (α2).

**Figure 6:**
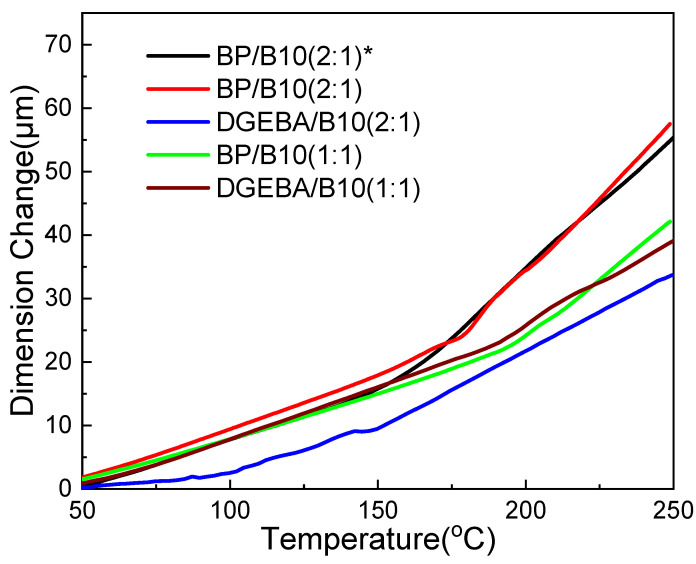
Thermal mechanical analysis of epoxy (*: anisotropic).

As seen from Table 4 and Figure 6, the anisotropic BP/B10 (2:1) had smaller values of α1 and α2, which suggests the existence of a more stable ordered LC structure and better dimensional stability. α1 and α2 of BP/B10 (2:1) were larger than DGEBA/B10 (1:1), because of the lack of a curing agent and the formation of a looser network. It can be seen that α1 of BP/B10 (1:1) was smaller than DGEBA/B10 (1:1), because the rigid-rod-like structure was not easy to move before Tg. The α2 of BP/B10 (1:1) was relatively large, because BP was easier to move after Tg, since it cured at lower temperatures for longer. The Tg of anisotropic BP/SAA (1:1) [20] was 200 °C, which was higher than anisotropic BP/B10 (2:1) but close to BP/B10 (1:1). The α1 and α2 of anisotropic BP/SAA (1:1) were 20.76 and 183.19. The α2 of anisotropic BP/B10 (2:1) was smaller than anisotropic BP/SAA (1:1), which demonstrated that the LC phase in anisotropic BP/B10 (2:1) provided a more stable structure than anisotropic BP/SAA (1:1) after Tg.

### 3.6. DEA Analysis

The dielectric constant could be reduced by the decrease in the dipole polarization in more highly crosslinked systems. The dielectric constant of the materials reduces with the increasing frequency when the frequency is lower than 10^4^ Hz. This is because the dipoles of the materials can keep up with the change of electric field at low frequency and the remaining polarization is strong [49].

The dielectric loss factor is also correlated to the crosslinking density from its effect on the lagging of dipole polarization. The dielectric loss of the materials increases with the increasing frequency (10^2^–10^6^ Hz), and shows a maximum in the range of 10^4^–10^5^ Hz. Due to the effect of internal viscous and friction forces, the dipole polarization will absorb lots of electric field energy and transfer it to heat, resulting in an increase in dielectric loss [50]. When the electric field frequency increases, the electric field energy consumed by dipole polarization also rapidly increases [51,52]. Therefore, the dielectric loss increases as the frequency of the electric field increases. However, as with the dependence of the dielectric constant on the frequency of the electric field, if the frequency of the electric field is high enough (>10^5^ Hz), the dipoles inside the material will maintain the relaxation process because they do not have enough time to establish polarization. In this case, the electric field energy consumed to overcome internal viscous and frictional forces is reduced, and this change is manifested as a reduction in dielectric loss [52,53,54].

**Figure 7:**
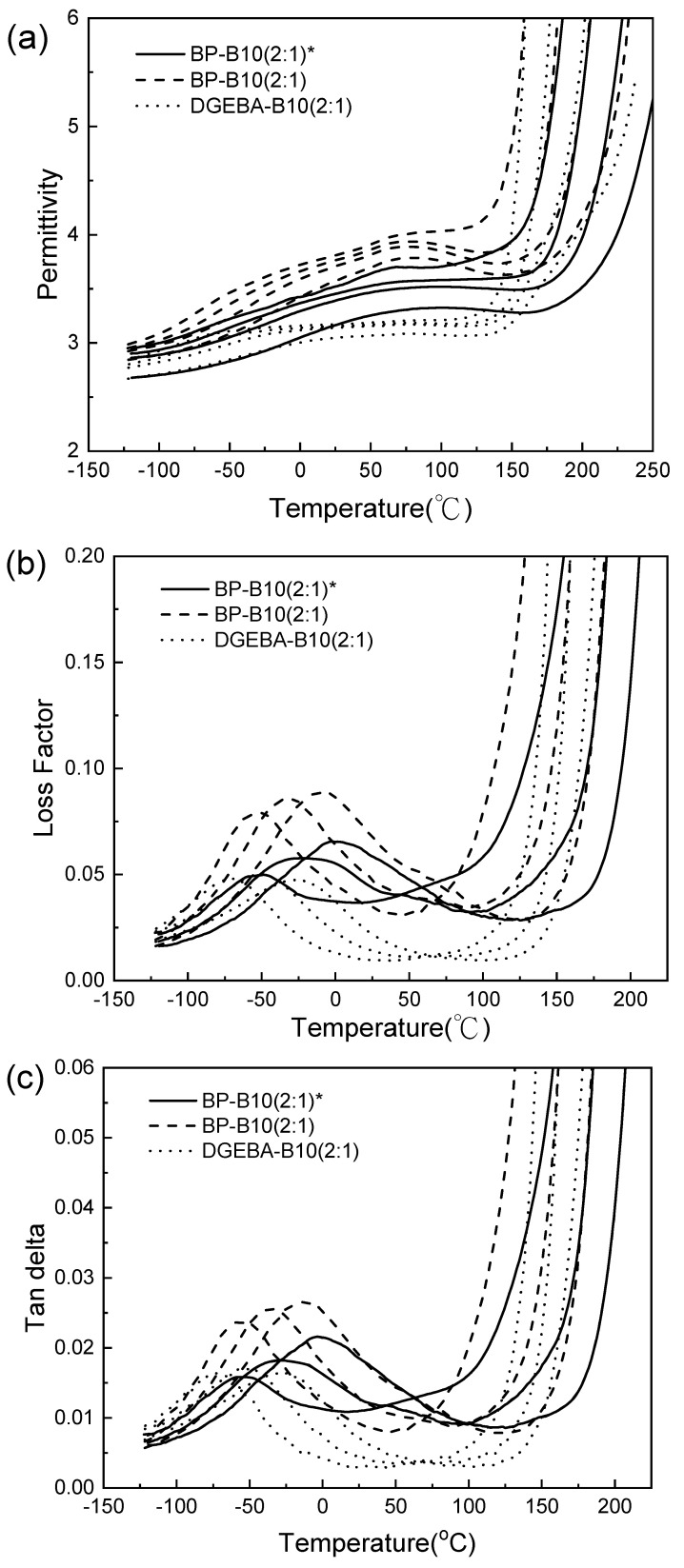
(**a**) Permittivity, (**b**) loss factor and (**c**) tan delta of cyanate ester (B10)-cured epoxy at 100, 1000 and 10,000 Hz (curves from left to right) (*: anisotropic).

As seen from Table 5, Table 6 and Table 7, and Figure 7a–c, the dielectric constant of anisotropic BP/B10 (2:1) was lower than that of BP/B10 (2:1), but it was higher than that of DGEBA/B10 (2:1), and the loss factor was the same. This is because the ratio of 2:1 leads to less curing agent. The BP system was more difficult to crosslink and had more linear chains, while the DGEBA system had a softer molecular chain, although there was less curing agent. However, it was easy to move and crosslink, and the degree of crosslinking was higher, so it had better dielectric properties than the BP system.

The lower β relaxation temperature of DGEBA/B10 means that only lower energy was required to move its short segments because of the softer structure of DGEBA. The β relaxation temperatures of the anisotropic BP/B10 (2:1) and BP/B10 (2:1) were slightly higher, which means that higher energy was required to move their short segments. This is because the BP structure was harder. The α relaxation of the BP/B10 and DGEBA/B10 systems was very large, which may be due to trapping of the main chain of small molecules that moved first.

**Figure 8:**
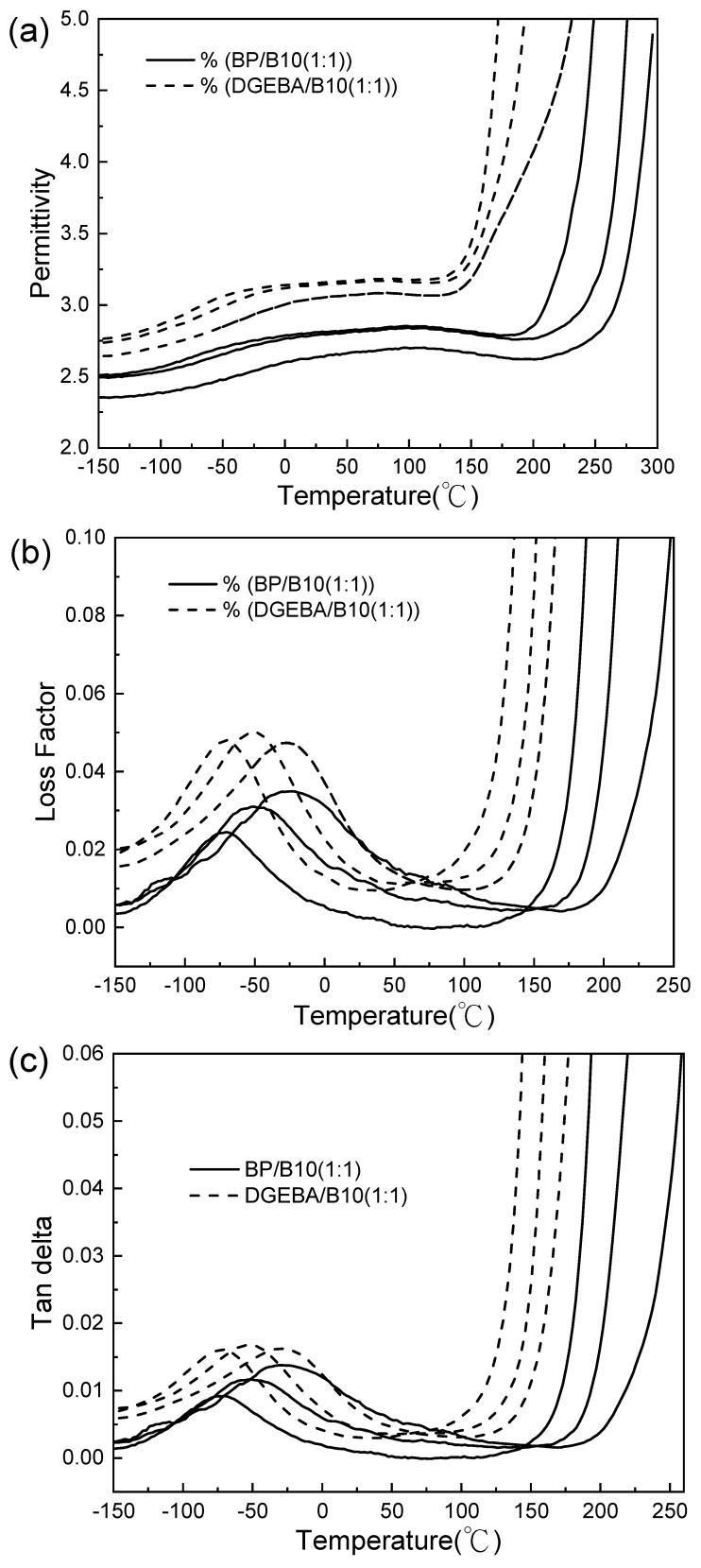
(**a**) Permittivity, (**b**) loss factor and (**c**) tan delta of cyanate ester (B10)-cured epoxy at 100, 1000 and 10,000 Hz (curves from left to right).

From Table 8 and Table 9, and Figure 8a–c, it is seen that BP/B10 (1:1) had a lower dielectric constant and loss coefficient than DGEBA/B10 (1:1). This is because the ratio of 1:1 provided enough curing agent, BP/B10 was easy to form a network structure and its dipole moment was not easy to move after formation, so the dielectric properties were better than DGEAB/B10. The anisotropic BP/B10 (2:1) and BP/B10 (1:1) showed better dielectric constant and dielectric loss than the report of DGEBA/B10 (1:1) [31]. BP/B10 (1:1) also had better dielectric properties than TMBP/B10 (2:1) and TMBP/B10 (1:1) [18], as well as DGEBA/B10 [52,55].

The plots of the dielectric loss vs. frequency were used to obtain the peak temperature and the corresponding frequency, as shown in Table 10. Plots of the logarithm of the frequency vs. the reciprocal of the temperature (log f vs. 1000/Tmax), and the apparent activation energy after linear regression, were obtained, as shown in Table 11. The activation energy of anisotropic BP/B10 (2:1) was higher than that of BP/B10 (2:1) and DGEBA/B10 (2:1). The activation energy of BP/B10 (1:1) was higher than that of DGEBA/B10 (1:1), but the activation energy of BP/B10 (1:1) was lower than that of anisotropic BP/B10 (2:1). Since the curing time of anisotropic BP/B10 (2:1) and BP/B10 (1:1) was longer, and anisotropic BP/B10 (2:1) may contain a LC phase after curing, higher energy of the side chain is required to move.

## 4. Conclusions

The thermal properties, including thermal gravimetric properties, dynamic mechanical properties and thermal expansion coefficient, and the dielectric properties of rigid-rod epoxy (BP) and commercial DGEBA epoxy, cured with cyanate ester (B10) as a curing agent, were studied. In terms of the thermal decomposition properties, anisotropic BP/B10 (2:1) had the highest 5% weight loss temperature and the highest amount of residue. DGEBA/B10 cracking residue was low, and the fastest cracking rate was large. Since anisotropic BP/B10 (2:1) had a longer curing time, it had time to form a more stable ordered network structure, and the BP rigid-rod chain structure was less likely to be broken into small molecules. With respect to the dynamic mechanical analysis, anisotropic BP/B10 (2:1) and BP/B10 (1:1) had higher Tg and harder network structure than other samples. Due to the long curing time at LC phase temperature, the BP system formed a relatively stable network structure, and its rigid-rod chain structure was not as soft as DGEBA. From dimensional stability analysis, anisotropic BP/B10 (2:1) had smaller α1 and α2, which suggests a more stable network structure and better dimensional stability. From dielectric analysis, the dielectric constant of anisotropic BP/B10 (2:1) was lower than that of BP/B10 (2:1), but higher than that of DGEBA/B10 (2:1) because there was less curing agent and the BP system was harder and more difficult to crosslink, while the DGEBA system had a softer molecular chain with more straight chains. Although DGEBA had less curing agent, it was easy to move and crosslink and had a higher degree of crosslinking, so it had better dielectric properties than the BP system. BP/B10 (1:1) had a lower dielectric constant than DGEBA/B10 (1:1). Since there was enough curing agent, BP/B10 was easy to form an isocyanurate network structure, curing time was long and its dipole moment was not easy to move after formation, so the dielectric properties were better than DGEBA/B10. The anisotropic BP/B10 system had better thermal properties than the commercial epoxy with flexible structure. BP/B10 also demonstrated better dielectric properties than the commercial epoxy resin when enough curing agent was provided.

## Figures and Tables

**Table 1:** 5% weight loss temperature and residual weight of cyanate ester-cured epoxy resin.

Cured Epoxy	Epoxy:B10 Ratio	5% Weight Loss Temp (°C)	Residual Weight (%)			
			At 350 °C	At 400 °C	At 450 °C	At 500 °C
BP *	2:1	362.7	96.65	79.85	45.26	40.30
BP		304.4	74.62	46.48	28.77	26.49
DGEBA		344.3	94.31	65.62	16.80	14.86
BP	1:1	335.1	92.35	62.53	42.77	38.96
DGEBA		344.7	94.21	60.13	34.40	21.23

*: Anisotropic.

**Table 2:** Highest rate of weight loss temperature and derivative weight of cyanate ester (B10)-cured epoxy resin.

Cured Epoxy	Epoxy:B10 Ratio	Highest Rate of Weight Loss Temp (°C)	Deriv. Weight at the Highest Rate of Weight Loss Temp (%/°C)
BP *	2:1	414.3	0.93
BP		400.7	0.68
DGEBA		418.5	1.57
BP	1:1	392.2	0.77
DGEBA		394.3	1.40

*: Anisotropic.

**Table 3:** Glass transition temperature of cured epoxy resin.

Cured Epoxy	Epoxy:B10 Ratio	Peak Value of DMA’s tanδ (Tg, °C)
BP *	2:1	190
BP		183
DGEBA		184
BP	1:1	253
DGEBA		223

*: Anisotropic.

**Table 4:** Tg and thermal expansion coefficients of cyanate ester-cured resin.

Cured Epoxy	Epoxy:B10 Ratio	Tg (°C)	CTE (μm/m°C)	
			α1	α2
BP *	2:1	174.2	60.6	150
BP		179.5	80.0	218
DGEBA		149.8	65.4	171
BP	1:1	197.3	73.8	182
DGEBA		202.5	85.9	134

*: Anisotropic.

**Table 5:** Permittivity and loss factor of cured resin at 10, 100, 1000 and 10,000 Hz, and −150, 30 and 150 °C, at BP:B10 = 2:1 (anisotropic).

Frequency (Hz)	Temperature (°C)	Permittivity	Loss Factor
10	−125	2.597	0.012
	30	3.707	0.263
	150	3.497	0.620
100	−125	2.575	0.009
	30	3.451	0.088
	150	3.252	0.141
1000	−125	2.555	0.010
	30	3.374	0.054
	150	3.150	0.060
10,000	−125	2.465	0.009
	30	3.334	0.066
	150	3.014	0.038

**Table 6:** Permittivity and loss factor of cured resin at 10, 100, 1000 and 10,000 Hz, and −150, 30 and 150 °C, at BP:B10 = 2:1.

Frequency (Hz)	Temperature (°C)	Permittivity	Loss Factor
10	−125	2.929	0.024
	30	3.826	0.064
	150	4.819	0.016
100	−125	2.903	0.613
	30	3.781	0.045
	150	3.929	0.013
1000	−125	2.884	0.110
	30	3.728	0.033
	150	3.749	0.012
10,000	−125	2.811	0.046
	30	3.602	0.029
	150	3.632	0.011

**Table 7:** Permittivity and loss factor of cured resin at 10, 100, 1000 and 10,000 Hz, and −150, 30 and 150 °C at DGEBA:B10 = 2:1.

Frequency (Hz)	Temperature (°C)	Permittivity	Loss Factor
10	−125	2.837	0.028
	30	3.172	0.020
	150	4.037	1.856
100	−125	2.797	0.024
	30	3.155	0.013
	150	3.431	0.312
1000	−125	2.765	0.023
	30	3.142	0.010
	150	3.312	0.074
10,000	−125	2.072	0.018
	30	3.051	0.011
	150	3.184	0.032

**Table 8:** Permittivity and loss factor of cured resin at 10, 100, 1000 and 10,000 Hz, and −125, 30 and 150 °C at BP:B10 = 1:1.

Frequency (Hz)	Temperature (°C)	Permittivity	Loss Factor
10	−125	2.548	0.005
	30	2.821	0.020
	150	2.833	0.040
100	−125	2.525	0.284
	30	2.812	0.011
	150	2.814	0.006
1000	−125	2.505	0.044
	30	2.795	0.002
	150	2.802	0.004
10,000	−125	2.362	0.009
	30	2.638	0.001
	150	2.665	0.040

**Table 9:** Permittivity and loss factor of cured resin at 10, 100, 1000 and 10,000 Hz, and −125, 30 and 150 °C in DGEBA:B10 = 1:1.

Frequency (Hz)	Temperature (°C)	Permittivity	Loss Factor
10	−125	2.802	0.016
	30	3.064	0.008
	150	3.075	0.035
100	−125	2.757	0.014
	30	3.048	0.011
	150	3.052	0.013
1000	−125	2.756	0.014
	30	3.031	0.015
	150	3.038	0.011
10,000	−125	2.652	0.014
	30	2.943	0.020
	150	2.963	0.008

**Table 10:** Sub-glass transition temperature of cyanate ester-cured epoxy resin at 2:1 and 1:1 at different frequencies.

Cured Epoxy	10 Hz	100 Hz	1000 Hz	10,000 Hz
BP/B10 (2:1) *	−85.08	−55.45	−23.64	1.57
BP/B10 (2:1)	−70.09	−50.19	−34.27	−6.44
DGEBA/B10 (2:1)	−82.29	−70.20	−49.95	−29.58
BP/B10 (1:1)	−87.74	−70.35	−50.60	−21.05
DGEBA/B10 (1:1)	−77.79	−61.95	−41.80	−21.89

*: Anisotropic.

**Table 11:** Apparent activation energy of cyanate ester-cured epoxy resin.

Cured Epoxy	Apparent Activation Energy (KJ/mol)
BP/B10 (2:1) *	61.78
BP/B10 (2:1)	44.54
DGEBA/B10 (2:1)	42.33
BP/B10 (1:1)	51.88
DGEBA/B10 (1:1)	42.17

*: Anisotropic.
